# A high definition Mueller polarimetric endoscope for tissue characterisation

**DOI:** 10.1038/srep25953

**Published:** 2016-05-12

**Authors:** Ji Qi, Daniel S. Elson

**Affiliations:** 1 Hamlyn Centre for Robotic Surgery, Institute of Global Health Innovation, Imperial College London, Exhibition Road, London, SW7 2AZ, UK; 2 Department of Surgery and Cancer, Imperial College London, Exhibition Road, London, SW7 2AZ, UK

## Abstract

The contrast mechanism of medical endoscopy is mainly based on metrics of optical intensity and wavelength. As another fundamental property of light, polarization can not only reveal tissue scattering and absorption information from a different perspective, but can also provide insight into directional tissue birefringence properties to monitor pathological changes in collagen and elastin. Here we demonstrate a low cost wide field high definition Mueller polarimetric endoscope with minimal alterations to a rigid endoscope. We show that this novel endoscopic imaging modality is able to provide a number of image contrast mechanisms besides traditional unpolarized radiation intensity, including linear depolarization, circular depolarization, cross-polarization, directional birefringence and dichroism. This enhances tissue features of interest, and additionally reveals tissue micro-structure and composition, which is of central importance for tissue diagnosis and image guidance for surgery. The potential applications of the Mueller polarimetric endoscope include wide field early epithelial cancer diagnosis, surgical margin detection and energy-based tissue fusion monitoring, and could further benefit a wide range of endoscopic investigations through intra-operative guidance.

Endoscopes are pivotal devices for non-invasive diagnostic investigation and minimally invasive image guided surgery, and novel image contrast mechanisms that can improve tissue characterization as well as specificity and sensitivity of detection are highly desirable. The overwhelming majority of medical endoscopes detect contrast from the fundamental interactions between light and biological tissue, inclusive of absorption, elastic scattering, and increasingly commonly, fluorescence. In essence, the images are obtained based on the fundamental properties of Electro-Magnetic waves, namely, the radiation intensity and wavelength. Another fundamental property of light, the polarization, can not only reveal tissue scattering and absorption information from a different perspective, but is also able to provide a fresh insight into directional tissue birefringence properties induced by anisotropic micro-structures such as collagen and elastin, the changes of which are significant pathological features. Therefore, if an endoscope could acquire the complete polarization properties of tissue, an additional contrast mechanisms would be realised and become available for tissue diagnosis and surgical treatment.

Biological tissue is highly scattering and incident polarized photons that are singly scattered or undergo a small number of scattering events near the tissue surface maintain this polarization state, while those multiply scattered penetrate deeper and are depolarized. This principle has been applied to select single-scattering spectra from multiply-scattered backgrounds for cell nucleus and cytoplasm size characterization^1^ and for *in situ in vivo* early cancer diagnosis^2^. This approach can reveal features at superficial depths such as blood vessels for microcirculation imaging^3^, or to increase image contrast near the tissue surface^4^. A few endoscopes that can resolve linear polarization states were also designed and demonstrated^2^,^5^,^6^ by using a pair of parallel and orthogonal linear polarizers for detection with respect to illumination polarization. This type of approach can only be applied to isotropic tissues that do not demonstrate any birefringence, dichroism or anisotropic linear depolarization, because linear depolarization images for anisotropic tissues are dependent on the sample orientation and the incident polarization direction resulting in bad reproducibility. They also lack of the ability to the properties of image circular depolarization, dichroism and birefringence.

Birefringent anisotropic structures and compositions are widely found in many tissue types^7^. One example tissue component is collagen, a major structural protein found in the extracellular matrix. The detection collagen structural content and organisation is of great importance for diagnosis of a variety of diseases including cancer^8^. Normal endoscopes that are insensitive to polarization and endoscopes that detect linear depolarization only are not able to reveal useful tissue birefringence information. To date, the most feasible birefringence-compatible endoscopic techniques are based on polarization sensitive optical coherence tomography (PS-OCT), but these are unable to detect depolarization properties due to the fundamental limitations of interferometry^9^,^10^. The point by point nature of the acquisition also limits its application in clinical practice due to the challenges in rapid acquisition and miniaturisation.

A Mueller matrix is a complete mathematical characterization of an object’s polarization properties. All the primary polarization characteristics, including linear depolarization, circular depolarization, directional birefringence, optical rotation and diattenuation etc., can be quantitatively interpreted from a Mueller matrix^11^,^12^. Mueller polarimetric imaging records spatially-resolved Mueller matrices over a field of view, which is emerging as a novel tissue imaging technique with potential to i) detect early cancer^13^,^14^,^15^,^16^, ii) to evaluate cancer progression^17^, iii) to evaluate residual cancerous tissue after radio/chemo-therapy^18^, iv) to monitor local structural disorders of the bladder wall caused by partial bladder outlet obstruction^19^, v) to accelerate or replace pathology^16^,^20^,^21^,^22^, vi) to assess tissue microstructures^22^,^23^,^24^ etc. However, Mueller polarimetric imaging has not become an endoscopic imaging technique, to the best of our knowledge, due to the lack of a practical and clinically feasible design.

Progress in constructing an endoscope suitable for partial Mueller polarimetric imaging that can convey 9/16 of the complete Mueller matrix has been reported^25^, but does not contain information related to circular polarization including circular depolarisation, circular diattenuation. These parameters have turned out to be useful^26^,^27^,^28^,^29^ and can be obtained using complete 4 × 4 Mueller polarimetry rather than the partial Mueller polarimetry. Additionally, the interpretation method for the 3 × 3 Mueller matrix proposed in^30^ requires a number of assumptions including known circular diattenuation and isotropic depolarisation for orthogonal linear polarisation. If these assumptions cannot be fully satisfied, there would be discrepancy between the decomposition results extracted based on 3 × 3 Mueller matrix decomposition^30^ and widely used 4 × 4 Mueller matrix polar decomposition^11^, according to^31^. Probing the Mueller matrix through a single mode fibre (SMF) was also demonstrated with the assumption that the SMF without any polarization dependant loss was equivalent to a retarder with constant phase retardance^32^. The assumption is invalid in endoscopic environments because the phase retardance of SMF changes acutely with bending, twisting and temperature^33^. As a point-measurement technique, acquisition times for individual measurements are not sufficiently short and would take at least 1.94 hours to reconstruct one image with one million pixels. Additionally it is highly challenging to miniaturise a switchable mirror^32^ and a fast raster-scanner so as to translate this point-measurement technique to wide field imaging.

Here we demonstrate a wide field high definition Mueller polarimetric endoscope (MPE) with minimal alteration to a rigid endoscope. The MPE inherits the advantages of rigid endoscopes with long life, a large range of working distances and low chromatic aberrations, in addition to high definition, wide field of view, and easy adaption to standard endoscope light sources as well as endoscopic imaging modalities such as narrow band imaging and fluorescence imaging. By imaging birefringent tissue mimicking phantoms and a porcine bladder, we show that this novel endoscopic imaging modality is able to provide a number of image contrast mechanisms besides traditional unpolarized radiation intensity, including linear depolarization, circular depolarization, cross-polarization, directional birefringence and dichroism. The information contributes to enhancing tissue features, and reveals tissue micro-structures. The potential applications of the MPE can benefit many endoscopic investigations or provide intra-operative guidance, including for early epithelium cancer diagnosis, surgical margin detection, energy-based tissue fusion monitoring.

## Results

### Mueller polarimetric endoscope system design and calibration

**Figure 1:**
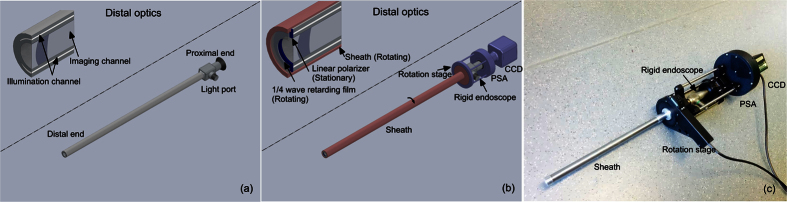
(**a**) A rigid endoscope consists of an imaging channel and an illumination channel. (**b**) The Mueller polarimetric endoscope consists of a stainless steel sheath, a motorised rotation stage, a rigid endoscope, a polarization state analyser (PSA) and a CCD image sensor. The part that rotates during acquisition is represented in red, and the stationary part is in purple. (**c**) A photo of the Mueller polarimetric endoscope.

States of light polarization (SOPs) are characterised by four-element Stokes vectors. A complete description of the transformation of SOPs during light matter interactions is thus a 4 × 4 (Mueller) matrix. Besides a light source and an image sensor, a Mueller polarimetric imaging system consists of a polarization state generator (PSG) to generate the required SOPs of incident light corresponding to minimally four linear independent Stokes vectors and a polarization state analyser (PSA) to analyse SOPs of emergent light. PSGs and PSAs have an exact equivalent configuration, and in free-space implementations, they usually comprise of a combination of rotatable and removable polarizing components or variable retarders made of liquid crystals or electro-optical crystals. Both the illumination and imaging channels of an ideal endoscope suitable for Mueller polarimetric imaging would be perfectly polarization maintaining so that the design of free-space PSGs and free-space PSAs could be simply duplicated at the proximal ends of both the channels. However, illumination channels (Fig. 1(a)) comprise of incoherent fibre bundles made up of individual large core diameter optical fibres - for wide and uniform spatial distribution and high transmission of luminance as well as good coupling efficiency at the input light port - which thoroughly scramble input SOPs.

Considering that miniaturisation of a free-space PSG is not technically feasible, we designed an endoscope sheath made of stainless steel with one end mounted to a motorised rotation stage near the proximal end of the rigid endoscope, as displayed in Fig. 1(b). At the distal end of the sheath, a 1/4 wave retarding film was mounted. As a result, the rotation could be transmitted by the sheath from the motorised rotation stage near the proximal end of the rigid endoscope to the retarding film at its distal end. The distal end of the illumination channel of the rigid endoscope was bonded to a linear polarizing film and kept stationary with respect to the PSA and the image sensor (a CCD in this work). Both the retarding and linear polarizing films were ring-shaped to avoid interference with the endoscope imaging channel. The stationary linear polarizing film and the rotating retarding film constituted an endoscopic PSG. The polarization axis of the stationary linear polarizing film was defined as 90°, and the fast axis orientations of the rotatable retarding film during one complete acquisition of a Mueller polarimetric image were selected as −45°, 0°, 30° and 60° so that four optimised polarization states were sequentially generated^34^ via rotating the sheath. The information about the manufacturer and model of the components used for the MPE are listed in Methods.

Although the imaging channels of rigid endoscopes may be birefringent^35^, it is still possible to utilise a free-space PSA at the proximal end of the rigid endoscope if the phase retardance induced by the imaging channel is calibrated. Calibration is also an essential consideration for Mueller polarimetry because orientations of linear polarizers and retarders in PSGs and PSAs are difficult to control precisely and the phase retardance is sensitive to wavelength bands and imaging geometry thus normally deviating from their nominal values. Here we proposed a two-step method to calibrate the MPE that works in a reflection mode. In the first step, an external free-space PSG (un-calibrated) was employed. The imaging channel of the rigid endoscope and the free-space PSA contained in the MPE were effectively considered as an “endoscopic PSA”. It is noted that the imaging channel is a phase retarder so it does not affect the optimised setting of a free-space PSA. The external PSG and the endoscopic PSA constitute a Mueller polarimetric imaging system in transmission mode. The eigenvalue calibration method^36^ was used to reverse the instrument matrices of the external PSG and the endoscopic PSA simultaneously. In the second step, the external PSG was removed, and a silver mirror which is polarization maintaining was used to calculate the instrumental matrix of the endoscopic PSG. The constructed Mueller polarimetric endoscope was validated by imaging a linear polarizer and a circular polarizer on top of a highly depolarization-dominant paper target as well as the phantom experiment shown in the next section. The typical Mueller matrix elemental errors are smaller than 7% and are mainly systematic. The validation results (the 4 × 4 Mueller polarimetric images of the polarisers) are provided in Supplementary information 1.

The angle of view of this MPE is 60° and could further reach the angle of view of the rigid endoscope by optimising the optics between the endoscope eyepiece and the image sensor. The spatial resolution of the MPE is mainly determined by the pixel size of the image sensor employed. The digital image resolution is determined by the pixel number of the image sensor which is 1.4 million in this work and is over the 0.92 million pixels for 720p HD display. Hence, this polarimetric endoscope is suitable for conventional high definition imaging.

### Tissue mimicking phantom studies

**Figure 2:**
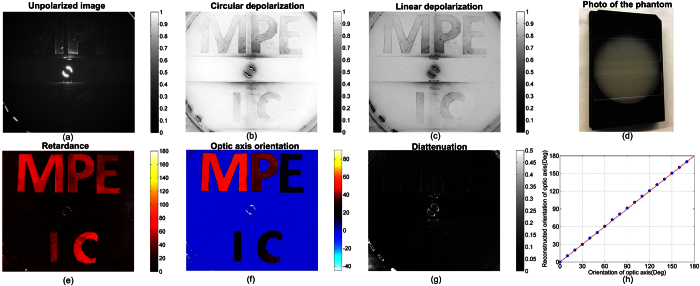
Results of the tissue mimicking phantom experiment. (**a**) unpolarized image (showing an area about 6.5 × 6.5 cm^2^), (**b**) circular depolarization image, (**c**) linear depolarization image, (**d**) a photo of the phantom using a normal camera, (**e**) phase retardance image, (**f**) optic axis orientation image, (**g**) diattenuation image, (**h**) The top layer of the phantom was replaced with a quarter wave retarder rotated from 0° to 170° with a step of 10°. The horizontal and vertical axes are actual and measured orientation of optic axis of the quarter wave retarder. The fast axis orientations detected by the MPE are represented by the blue circle and are distributed around the predicted red diagonal line.

The backscattered non-depolarized light can act as a probe to detect tissue birefringence and dichroism, and highlight the absorption and scattering within the non-depolarized (superficial) volume of tissue. Accordingly, a tissue mimicking phantom with two layers was constructed to assess the imaging ability of MPE. A layer was prepared with 2 ml 10%-intralipid and 0.2 ml India ink dissolved in 100 ml distilled water to simulate tissue scattering and absorption at 546 nm respectively. The scattering properties about this layer can be found in^37^. A top layer was made to simulate the tissue birefringent structures and compositions within the non-depolarized volume for incident polarized light by cutting out five letters “M” “P” “E” “I” “C” from a piece of a transparent birefringent plastic film that was equivalent to a phase retarder. The letters were clamped between two microscopic glass slides as displayed in Fig. 2(d). The optic axis orientations of “P” and “E” in the first row were 15° rotated from their left letter individually, and the optic axis orientations of “I” and “C” were the same. The Mueller polarimetric images of the phantom were acquired by the MPE. The working distance of the MPE for this experiment was about 8 cm. The regions near the edges of the field of view were empty and were therefore cropped: Fig. 2(a–c, e–f) each showed 0.75 million pixels out of 1.4 million pixels and showed an area about 6.5 × 6.5 cm^2^. The elemental image in the first row and column of the 4 × 4 Mueller matrix images *m*_11_ corresponds to unpolarized radiometric imaging^38^ and was used to compare with the reconstructed polarization images. The Mueller matrix images were then divided by *m*_11_ to generate a normalised form of Mueller matrix which were demonstrated in Supplementary information 1. The normalised Mueller matrix images were interpreted based on the polar decomposition method^11^ to obtain the specific polarization properties of interest, namely, linear depolarization, circular depolarization, diattenuation (quantifying dichroism) as well as phase retardance and the optic axis orientation of retardance that quantitatively represents directional birefringence, and are displayed in Fig. 2.

Although scattering from parts of the randomly orientated micro-facets of the rough letter edges helped to visualise some of the letters in the unpolarized image, as shown in Fig. 2(a), it was difficult to differentiate the letters from the substrate by unpolarized imaging. The linear depolarization of the substrate layer (about 0.85) was lower than its circular depolarization (about 0.98), demonstrated in Fig. 2(b,c). This trend in the turbid media was also reported in the literature^17^,^39^. The substrate layer of the phantom was isotropic in terms of refractive index, so it was only depolarizing, and demonstrated negligible diattenuation and phase retardance. The optic axis orientation of practically zero phase retardance was not a number and was assigned as 0° in Fig. 2(f). In the phase retardance image in Fig. 2(e), five birefringent letters were remarkably different from the non-birefringent substrate. Since the letters were made from the same piece of birefringent film, they showed a similar phase retardance value. The optic axis orientations of the letters are quantitatively visualised in Fig. 2(f) and were in accordance to the expectation of the 15° rotation between the “M” “P” “E” letters and the same orientation for the “I” and “C”.

The diattenuation of the letters in Fig. 2(g) was as weak as the substrate because the letters were not dichroic, but was slightly enhanced at the letter edges because the micro-facets singly scattered the light resulting in a higher diattenuation. Therefore, this phantom study demonstrated that the MPE is able to detect birefringence in the non-depolarized volume of turbid media in a reflection geometry. The reconstructed images showing phase retardance and the optic axis orientation demonstrate obvious advantages to the unpolarized image when considering turbid media birefringence.

To further illustrate the ability of the MPE to resolve the optic axis of phase retardance, the top layer of the phantom was replaced with a quarter retarder (WPQ10E-546, Thorlabs Ltd. Ely, UK, phase retardance characterised as 83°). The retarder was sequentially rotated from 0° to 170° with a step of 10° and imaged using the MPE. The unpolarized radiometric intensity remained the same during rotation with a value 0.929 ± 0.013, as expected. Nevertheless, the MPE was able to reveal the orientations of the optic axis of the top layer of the phantom, as represented by the blue circles in Fig. 2(h) which are distributed around the predicted line indicated in red (the step size was measured as 9.9951° ± 0.5472°, close to the actual value of 10°). The phase retardance was measured to be 165.931° ± 5.047° as the non-depolarized backscattered light returns through the retarding film resulting in a double pass geometry. The linear depolarization and circular depolarization were essentially the same during the rotation of the top retarder layer (0.949 ± 0.005 and 0.831 ± 0.004 respectively) since the change of optic axis did not interfere with the scattering and absorption in the substrate. The diattenuation was as low as 0.029 ± 0.004 and also did not change very much with rotation. This experiment indicated that the MPE can accurately resolve the optic axis of phase retardance.

### *Ex vivo* tissue study

Experiments were performed on a porcine bladder to demonstrate that the MPE can reveal features that are either difficult to resolve or not resolvable for unpolarized endoscope by providing additional image contrast mechanisms including linear depolarization, circular depolarization and directional birefringence. The working distance of the MPE for this experiment was about 8 cm. The figures demonstrated in this section showed 1.07 million pixels out of 1.4 million pixels and showed an area about 7.8 × 7.8 cm^2^. The bladder was continuously distended using two clamps, one of which was fixed while the other one was mounted on a linear translation stage oriented away from the first clamp. The displacement of the linear translation stage ranged from 0 to 28 mm with a step size of 1 mm and MPE images were recorded for each step. The acquired Mueller polarimetric images shown in Supplementary information 1 were decomposed into specific polarization images based on polar decomposition^11^. The decomposed results are shown in a Supplementary video (with the video legend in Supplementary information 1) and two representative sets of the results without (displacement of 0 mm) and with distention (displacement of 15 mm) are displayed in this section. Regions that were saturated in at least one of the raw images used to reconstruct Mueller polarimetric images were invalid and are enclosed by the blue lines in the figures shown in this section.

**Figure 3:**
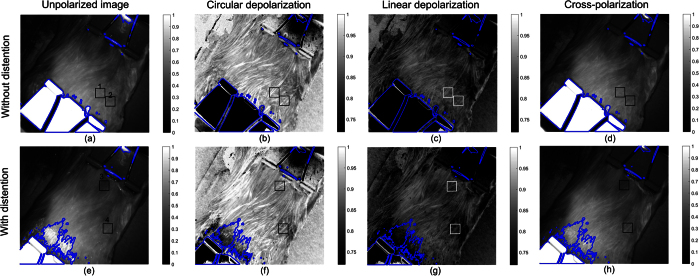
The images from the first to the fourth column are respectively unpolarized images, circular depolarization images, linear depolarization images, and cross polarization images of the bladder. Each image showed an area about 7.8 × 7.8 cm^2^. (**a–d**) are the images obtained when the bladder was not distended and (**e,f**) are those obtained when the bladder was under distention. Two regions on the bladder named ROI1 and ROI2 in (**a–d**) and two regions named ROI3 and ROI4 in (**e–h**) indicated with rectangular boxes were used for further analysis. Regions enclosed by the blue lines were invalid due to pixel saturation in at least one of the raw images for Mueller polarimetric image reconstruction.

The circular depolarization shown in Fig. 3(b) was higher than the linear depolarization in Fig. 3(c) overall, which is similar to the trend of the tissue mimicking phantom illustrated in the last section as well as many biological tissues including porcine fat, tendon, artery, myocardium^29^, liver, tendon, kidney cortex, brain, myocardium muscle, and loin muscle^40^, human skin basal cell carcinoma^15^, human colon^39^, etc. Larger circular depolarization than linear depolarization observed here is typically associated with Rayleigh-like scattering^29^,^39^,^40^, resulting from scattering by the particles in tissue much smaller than light wavelength like cell organelles^41^, as well as the particles the sizes of which are comparative to or larger than light wavelength but the relative refractive indices of which are close to unity^42^. Therefore, the relative comparison between linear and circular depolarisation may be used to reveal information about tissue scattering^29^,^39^,^40^, particularly, about the size and relative refractive index of scatterers^26^,^28^. It is noted that the absolute values of linear and circular depolarization are affected by both tissue absorption and scattering represented by absorption coefficients, scattering coefficients and scattering anisotropy^25^,^40^,^43^,^44^,^45^. Spatially random orientated tissue absorbers in bulk tissue attenuate the linearly and circularly polarised light without preference, and do not influence the characteristic length of depolarisation for linearly and circularly polarised light^46^, and therefore the ratio between linear and circular depolarisation should be less dependent on tissue absorption. The difference between tissue circular and linear depolarization obtained by the MPE can help to investigate tissue scattering, and how to use the circular and linear depolarisation information to quantify the scatter size and reduce the influence of absorption still requires further studies.

The circular depolarization image and the linear depolarization image were correlated. Both images demonstrated enhanced visualisation of superficial structures on the serosa of the bladder compared to the unpolarized image shown in Fig. 3(a). Epithelial cancer makes up about 85% of all cancers^47^. It is therefore important to enhance image information of the superficial epithelial tissue volume to detect early epithelial cancers. Figure 3(d) shows the cross-polarization image conveying information from deeper tissue, because this image was mainly generated from multiply-scattered deeper penetrating photons. The cross-polarization image can be used to identify micro-vascular structures of interest^3^. In comparison, the unpolarized technique convoluted the information from the superficial and deeper parts and therefore cannot achieve as good image contrast as depolarization imaging and cross-polarization imaging.

The influence of distention on the polarization properties of bladder can be assessed by comparing the images in the first and second row of Fig. 3. Circular depolarization in Fig. 3(f) is still higher than linear depolarization in Fig. 3(g), because distension did not change the dominance of Rayleigh scattering. The distention did not cause a significant change on the linear depolarization properties which can be observed from Fig. 3(c,g), but led to a subtle rise of circular depolarization, in particular for the upper left region that can be observed between Fig. 3(b,f). It is difficult to observe differences resulting from the distention in the unpolarized images (Fig. 3(a,e)) and cross-polarization images (Fig. 3(d,h)) except the displacement due to the stretch.

**Figure 4:**
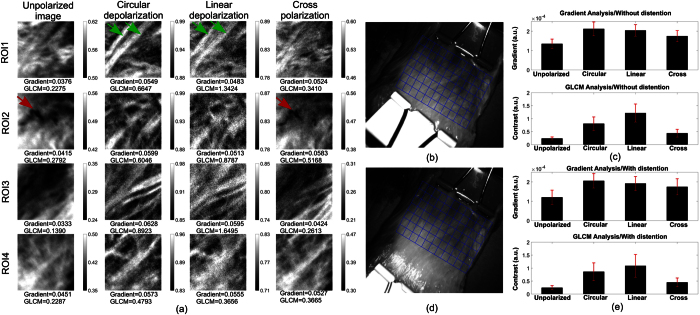
(**a**) The first to the fourth column present the unpolarized, circular depolarization, linear depolarization and cross-polarization images of the four sub-regions named ROI 1–4 indicated by the squares in Fig. 3. Two superficial diagonal linear (fibrous) structures in ROI1 are labelled with green arrows and a deeper blood vessel in ROI2 is indicated by a red arrow. The image contrast values calculated from image gradient magnitudes and grey-level co-occurrence matrix are displayed on the bottom of each sub-image correspondingly. The images of the bladder without distention in (**b**), and with distention in (**d**) were individually segmented into 77 sub-regions indicated by the 7 × 11 blue grid. The contrast was analysed for each sub-region by calculating image gradient and GLCM contrast of the sub-region. The charts of average image contrast for all the 77 sub-regions when the bladder was without distention in (**c**) and was with distention in (**e**). The upper graph was obtained from gradient analysis, and the bottom was the result of GLCM contrast analysis. The red error bar represents the standard deviation of image contrast data of the 77 sub-regions.

Two square sub-regions (80 × 80 pixels) on the bladder named ROI1 and ROI2 in Fig. 3(a–d) obtained with the non-distended bladder, and two square sub-regions (80 × 80 pixels) named ROI3 and ROI4 in Fig. 3(e–h) obtained with distention indicated were selected as examples to demonstrate that Mueller polarimetric endoscope is able to reveal features that are either difficult to resolve or not resolvable by unpolarized endoscope imaging. The unpolarized, circular depolarization, linear depolarization and cross-polarization data for these sub-regions are displayed in Fig. 4(a). It is noted that the range of the grey scale for each image in Fig. 4(a) was individually adjusted such that 0.5% of pixels is saturated at low and high grey levels of that image for an intuitive comparison, because unpolarised light intensity images and reconstructed depolarisation images have different physical meanings as well as different pixel value distribution. The contrast enhancement by Mueller polarimetric imaging was further explored by examining the image statistics of these datasets. Image contrast was quantitatively assessed by employing two metrics – the grey-level co-occurrence matrix analysis (GLCM)^48^ (a widely used method for texture contrast analysis in an image) and the mean value of image gradient magnitudes that can be used to denote sharpness of texture in the image. The image contrast values calculated with the two metrics are displayed below each sub-image in Fig. 4(a), and higher values are better.

In ROI1, two superficial diagonal linear (fibrous) structures labelled with green arrows can be more clearly observed in the circular and linear depolarization images than the unpolarized image. In terms of gradient analysis of ROI1, the circular depolarization provided the highest image contrast, and was 46% higher than the unpolarized image. The contrast in the linear depolarization image and the cross-polarization image increased by 28% and 39% respectively. The GLCM analysis of ROI1 demonstrated a similar trend with contrast increased by 192%, 490% and 50% for circular depolarization, linear depolarization and cross-polarization imaging compared to unpolarized imaging. In ROI2, the depolarization images visualised similar superficial fibre structures to those in ROI1 that cannot be resolved by unpolarized imaging. Although a deeper blood vessel indicated by a red arrow can be seen in the corresponding unpolarized image, the boundary of the vessel appeared sharper in the cross-polarization image. The image content of the cross-polarization image and depolarization images have little correlation, as the enhanced vessel in the cross-polarization image was entirely unobservable in the depolarization images. The image contrast of ROI2 was improved by 44%, 34% and 40% for circular depolarization, linear depolarization and cross-polarization respectively in terms of the mean image gradient, and 117%, 215% and 85% correspondingly for GLCM contrast analysis. In ROI3 and ROI4, similar contrast enhancement effects by the depolarization images and the cross-polarization images are also observed, and are in accordance to the rise of image gradient and GLCM contrast compared with that of the unpolarized images.

The images of the bladder without distention were then segmented into 77 sub-regions (55 × 55 pixels) indicated by the 7 × 11 blue grid in Fig. 4(b) for contrast analysis. The analysis showed that the contrast of the circular depolarization, the linear depolarization and cross-polarization images was almost always higher than the corresponding unpolarized image for all the individual sub-regions. The charts of the average image contrast of the 77 sub-regions are exhibited in Fig. 4(c), in which the upper graph was obtained from gradient analysis, and the bottom was the result of GLCM contrast analysis, and the red error bar represents the standard deviation of image contrast data of the 77 sub-regions. Circular depolarization and linear depolarization contained enhanced superficial information; cross polarization images can better visualise deeper tissue.

The quantitative analysis of image contrast for the bladder with distention was conducted in the same way. The images were segmented into 77 sub-regions shown by the blue grid in Fig. 4(d). As shown in the charts of the average image contrast displayed in Fig. 4(e), the contrast was enhanced by polarimetric images, and the enhancement trends are similar to those without distention in Fig. 4(c). In summary, all these depolarization and depolarization-related (cross-polarization) results suggest that by probing the information related to tissue depolarization, MPE can improve the visibility of superficial details of tissue. Deeper tissue features like blood vessels are enhanced by providing cross-polarization images, and therefore the MPE is superior to traditional unpolarized endoscopes.

Structures consisting of birefringent collagen and elastin, that traditional unpolarized endoscopes cannot visualise, have a critical role in detection of epithelial cancer^49^, obstructive disease of bladders^50^, etc. As was demonstrated in the phantom study, MPE was able to detect birefringence in non-depolarized (superficial) volumes of turbid media in reflection mode, while unpolarized imaging was not. Hence the MPE can provide a practical way for wide field high resolution imaging of collagen and elastin^51^ resulting from their anisotropic microstructures. The serosa of bladder imaged here is known to consist of mainly collagen and elastin fibrils according to second harmonic generation microscopy^19^ and electronic microscopy studies^52^.

**Figure 5:**
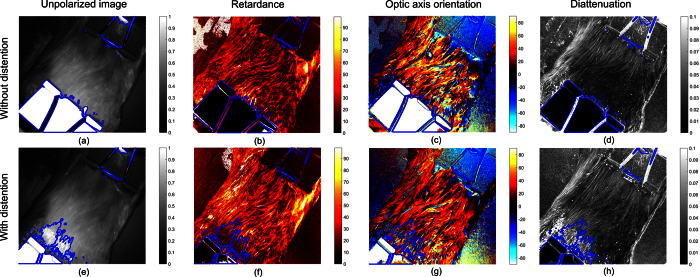
The images from the first to the fourth column are respectively unpolarized image, retardance image, optic axis orientation map, diattenuation images of the bladder. Each image showed an area about 7.8 × 7.8 cm^2^. (**a–d**) are the images obtained when the bladder was not under distention and (**e,f**) are those obtained when the bladder was under distention. Regions enclosed by the blue lines were invalid due to pixel saturation in at least one of the raw images for Mueller polarimetric image reconstruction.

The polarization images described above were further evaluated by creating images of the phase retardance and optical axis orientation, as shown in Fig. 5. When there was no distension, the phase retardance of the bladder serosa was successfully observed and appeared to be spatially varying – see Fig. 5(b). The orientations of optic axis across the entire image also spatially varied with most of the orientation values in the range from −40° to 80°, as exhibited in Fig. 5(c). After being distended, the phase retardance of the bladder displayed in Fig. 5(f) increased, and the optic axis orientations became more uniform across the image with values in the range from −30° to 40° exhibited in Fig. 5(g). A continuous increase in the phase retardance and the optic axis orientation uniformity with distention can be better seen in the Supplementary Video. This occurs because the spatially disordered anisotropic micro-structures of collagen and elastin become better aligned due to the applied one dimensional mechanical force in the distension process. These subtle changes were apparently not detectable by unpolarized imaging in Fig. 5(a,e).

It was also found that the increase in retardance that is observed in Fig. 5(f) was correlated with the increase in depolarization shown in Fig. 3(f), particularly for the upper left part of the bladder. This indicates that the rise of depolarization may be caused by rise of phase retardance, as corroborated by other studies^4^,^53^. The results illustrates that MPE is sensitive to the existence of birefringent structures of tissue and their subtle variations that are not resolvable by unpolarized endoscope imaging. Note that the diattenuation of the bladder was weak, as shown in Fig. 5(d,h), which is in accordance to many studies in tissue polarimetry^7^,^17^,^54^.

## Discussion

We have demonstrated a wide field high definition Mueller polarimetric endoscope with a minimally altered rigid endoscope system to reduce the cost and simplify the administrative approval procedures. It is compatible with existing infrastructure for minimally invasive surgery and robotic assisted surgery as well as techniques for narrow band imaging and fluorescence imaging. The associated design, calibration and image reconstruction procedures were also specified. By imaging birefringent tissue mimicking phantoms and a porcine bladder, we showed that this novel endoscopic imaging modality comprehensively characterized tissue polarization properties by measuring Mueller matrices, and thus provided a number of useful image contrast mechanisms besides traditional unpolarized radiation intensity, including linear depolarization, circular depolarization, cross-polarization, directional birefringence and dichroism. This information contributes to enhancing tissue features of interest, and additionally revealing tissue micro-structures, which is of central importance for tissue diagnosis and image guidance for surgery. Polar decomposition was adopted in this paper since it was introduced earlier into tissue polarimetry and is widely used in practice^7^,^14^,^19^,^40^. We note that considering tissues are layered structures, the emerging differential decomposition method for Mueller matrices may be closer to reality^55^. Whether differential decomposition offers a significant advantage is still under study in this field.

The potential applications of MPE can benefit the whole spectrum of endoscopic investigations and intra-operative guidance, including wide field early epithelial cancer diagnosis, surgical margin detection and tissue fusion monitoring.

## Methods

### The MPE setup

The rigid endoscope employed in this paper is a laparoscope of 0° viewing angle (HOPKINS II, Karl Storz GmbH & Co. KG, Tüttlingen, Germany) which is widely used to inspect and diagnose conditions or perform minimally invasive surgery mostly in the abdomen or pelvis through small incisions. The design is fully adaptable to the other types of rigid endoscopes. The light source was a commercial illumination system with a high pressure Mercury lamp (Lumen 200Pro, Prior Scientific Instruments Ltd. Cambridge, UK) and a band pass filter centred at 543 nm with 22 nm bandwidth (FF01-543/22-25, Semrock Inc. New York, US). A light guide delivered the light into the illumination channel of the rigid endoscope. The endoscope sheath was made based on a stainless steel tube with the external diameter 12.7 mm and internal diameter 10.9 mm, and was mounted on the motorized rotation stage (PRM1/MZ8, Thorlabs Ltd. Ely, UK) via a customized adapter. The ring-shaped retarding film and linear polarizer used at the distal end of the sheath was processed from a low cost polymer retarder film and polarizing laminated film with extinction ratio larger than 500:1 (Visible linear polarizing laminated film, Edmund Optics Ltd. York, UK). The retardance of the polymer retarder film at 543 nm is about 85°. The PSA was a motorized fast change filter wheel (FW103H/M, Thorlabs Ltd. Ely, UK) containing four linear polarizers with extinction ratio 9000:1 (High contrast plastic linear polarizer, Edmund Optics Ltd. York, UK) orientated at −45° 0° 45° 90° respectively and two circular polarizers (Edmund Optics Ltd. York, UK) with one left polarized and the other right polarized. The image sensor was a scientific CCD camera (Retiga-Exi, QImaging Co. Surrey, Canada). The shortest acquisition time the system can achieve is about 15 seconds per Mueller polarimetric image, limited by the slower PSG rotation stage. The acquisition time for the phantom and tissue experiments was about 30 seconds. Image processing would be useful to reduce the motion artefacts for the future *in vivo* studies. We are also developing a much faster Mueller polarimetric endoscope system based on a non-time sequential PSA and a PSG based on a faster motor to make acquisition in real time. Image registration for stacks of multispectral and polarization-resolved images acquired over similar time periods has previously been demonstrated using algorithms developed by ourselves and our collaborators^56^,^57^.

### Eigen value calibration

Calibration is a procedure to determine the real instrumental matrices of a PSG and a PSA, which normally deviate from their nominal expectations. It is an essential consideration in polarimetry. E. Compain *et al.* proposed the eigenvalue calibration method (ECM)^36^, which turned out reliable and general^58^,^59^,^60^.The ECM takes advantage of measuring calibration reference samples so as to obtain a proper group of system responses of the polarimeters. Normally, such a group of responses includes a null response, linear diattenuation responses and linear retardance responses to construct the real instrumental matrices of a PSG and a PSA at the same time. The ECM does not rely on any assumptions about the polarimeter operations or calibration samples. The retardance, diattenuation and the orientation of the calibration samples do not need to be precisely known. The ECM was used here to reverse the instrument matrices of the external PSG and the endoscopic PSA simultaneously. The calibration samples used were air, a linear polariser orientated at 0° (defined) and 90° (approxiamtely) and a quarter waveplate (phase retardance about 85°) with the fast axis orientated at about 30°.

### Polarization image reconstruction based on polar decomposition of Mueller matrix and the units of parameters

A physically realizable Mueller matrix can be decomposed into a sequence of three matrix factors representing depolarization *M*_∆_, retardance *M*_*R*_ and diattenuation *M*_*D*_ according to^11^, expressed by


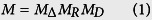


The principle depolarization factors are contained in the bottom right 3 × 3 sub-matrix *m*_∆_ of the depolarization matrix *M*_∆_^11^ which can be represented by^11^,


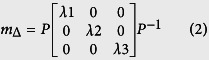


where *λ*_1_, *λ*_2_ and *λ*_3_ are the eigenvalues of *m*_∆_, and *P* is a matrix composed of the eigenvectors of *m*_∆_. (1 − *λ*_1_) and (1 − *λ*_2_) correspond to the depolarization values for two linear polarization states and the average of these two depolarization values ∆_*L*_ was used to reconstruct linear depolarization image. ∆_*L*_ (∆_*L *_= 1 − *λ*_3_) corresponds to depolarization for circular polarization states and was used for the reconstruction of circular depolarization image. ∆_*L*_and ∆_*C*_ range from 0 (corresponding to the non-depolarized) to 1 (referring to the fully depolarized) with arbitrary units.


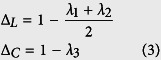


The retardance image was reconstructed from the trace of *M*_*R*_ as displayed in the following equation^11^.


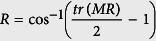


The range is from 0° to 180°. The orientation of optic axis ranging from −90° to 90° is calculated according to^19^


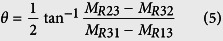


The diattenuation value *D* for the reconstruction of diattenuation image can be readily obtained from the second, the third and the fourth element in the first row of a Mueller matrix represented by *m*_12_, *m*_13_ and *m*_14_ respectively as shown in the following equation,


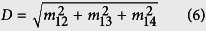


Polar decomposition was adopted in this paper since it is widely used into tissue polarimetry^7^,^14^,^19^,^40^. We note that considering tissues are layered structures, the emerging differential decomposition method for Mueller matrices may be closer to reality^55^.

### Tissue sample preparation

An *ex vivo* porcine urinary bladder was harvested from a Large White pig *post mortem*. The experiment in this manuscript did not involve any live animals. This pig was originally used for experiments conducted by a separate research group, and was terminated in accordance with UK Home Office animal licence regulations. A region about the middle 1/3 of the bladder between the ureter and the urethra was transacted, and lateral traction was applied to the open bladder with two clamps.

## Additional Information

**How to cite this article**: Qi, J. and Elson, D. S. A high definition Mueller polarimetric endoscope for tissue characterisation. *Sci. Rep.* **6**, 25953; doi: 10.1038/srep25953 (2016).

## Supplementary Material

Supplementary Video

Supplementary Information

## References

[b1] Backman V. *et al.* Polarized light scattering spectroscopy for quantitative measurement of epithelial cellular structures *in situ*. Selected Topics in Quantum Electronics, IEEE Journal of 5, 1019–1026 (1999).

[b2] Qiu L. *et al.* Multispectral scanning during endoscopy guides biopsy of dysplasia in Barrett’s esophagus. Nat. Med. 16, 603–606 (2010). 20383155 10.1038/nm.2138 PMC3052700

[b3] Groner W. *et al.* Orthogonal polarization spectral imaging: a new method for study of the microcirculation. Nat. Med. 5, 1209–1212 (1999). 10502828 10.1038/13529

[b4] Jacques S. L., Ramella-Roman J. C. & Lee K. Imaging skin pathology with polarized light. J. Biomed. Opt. 7, 329–340 (2002). 12175282 10.1117/1.1484498

[b5] Qi J., Barrière C., Wood T. C. & Elson D. S. Polarized multispectral imaging in a rigid endoscope based on elastic light scattering spectroscopy. Biomed. Opt. Express 3, 2087–2099 (2012). 23024903 10.1364/BOE.3.002087 PMC3447551

[b6] Clancy N. T. *et al.* Polarised stereo endoscope and narrowband detection for minimal access surgery. Biomed. Opt. Express 5, 4108–4117 (2014). 25574424 10.1364/BOE.5.004108 PMC4285591

[b7] Ghosh N. & Vitkin I. A. Tissue polarimetry: concepts, challenges, applications, and outlook. J. Biomed. Opt. 16, 110801 (2011). 22112102 10.1117/1.3652896

[b8] Chen P., Cescon M. & Bonaldo P. Collagen VI in cancer and its biological mechanisms. Trends Mol. Med. 19, 410–417 (2013). 23639582 10.1016/j.molmed.2013.04.001

[b9] Jiao S. & Wang L. V. Two-dimensional depth-resolved Mueller matrix of biological tissue measured with double-beam polarization-sensitive optical coherence tomography. Opt. Lett. 27, 101–103 (2002). 18007725 10.1364/ol.27.000101

[b10] Jiao S., Yao G. & Wang L. V. Depth-resolved two-dimensional Stokes vectors of backscattered light and Mueller matrices of biological tissue measured with optical coherence tomography. Appl. Opt. 39, 6318–6324 (2000). 18354641 10.1364/ao.39.006318

[b11] Lu S.-Y. & Chipman R. A. Interpretation of Mueller matrices based on polar decomposition. J. Opt. Soc. Am. A 13, 1106–1113 (1996).

[b12] Ghosh N., Vitkin I. A. & Wood M. F. G. Mueller matrix decomposition for extraction of individual polarization parameters from complex turbid media exhibiting multiple scattering, optical activity, and linear birefringence. J. Biomed. Opt. 13, 044036–044036 (2008). 19021363 10.1117/1.2960934

[b13] Chung J., Jung W., Hammer-Wilson M. J., Wilder-Smith P. & Chen Z. Use of polar decomposition for the diagnosis of oral precancer. Appl. Opt. 46, 3038–3045 (2007). 17514255 10.1364/ao.46.003038

[b14] Pierangelo A. *et al.* Polarimetric imaging of uterine cervix: a case study. Opt. Express 21, 14120–14130 (2013). 23787602 10.1364/OE.21.014120

[b15] Du E. *et al.* Mueller matrix polarimetry for differentiating characteristic features of cancerous tissues. J. Biomed. Opt. 19, 076013–076013 (2014). 10.1117/1.JBO.19.7.076013 25027001

[b16] He H. *et al.* Mapping local orientation of aligned fibrous scatterers for cancerous tissues using backscattering Mueller matrix imaging. J. Biomed. Opt. 19, 106007–106007 (2014). 25321399 10.1117/1.JBO.19.10.106007

[b17] Pierangelo A. *et al.* *Ex-vivo* characterization of human colon cancer by Mueller polarimetric imaging. Opt. Express 19, 1582–1593 (2011). 21263698 10.1364/OE.19.001582

[b18] Pierangelo A. *et al.* Multispectral Mueller polarimetric imaging detecting residual cancer and cancer regression after neoadjuvant treatment for colorectal carcinomas. J. Biomed. Opt. 18, 046014–046014 (2013). 23612875 10.1117/1.JBO.18.4.046014

[b19] Alali S. *et al.* Assessment of local structural disorders of the bladder wall in partial bladder outlet obstruction using polarized light imaging. Biomed. Opt. Express 5, 621–629 (2014). 24575354 10.1364/BOE.5.000621 PMC3920890

[b20] Wang W. *et al.* Investigation on the potential of Mueller matrix imaging for digital staining. J. Biophotonics 9, 364–375 (2015). 25907856 10.1002/jbio.201500006

[b21] He C. *et al.* Characterizing microstructures of cancerous tissues using multispectral transformed Mueller matrix polarization parameters. Biomed. Opt. Express 6, 2934–2945 (2015). 26309757 10.1364/BOE.6.002934 PMC4541521

[b22] Sun M. *et al.* Characterizing the microstructures of biological tissues using Mueller matrix and transformed polarization parameters. Biomed. Opt. Express 5, 4223–4234 (2014). 25574434 10.1364/BOE.5.004223 PMC4285144

[b23] He C. *et al.* Quantitatively differentiating microstructures of tissues by frequency distributions of Mueller matrix images. J. Biomed. Opt. 20, 105009–105009 (2015). 26502227 10.1117/1.JBO.20.10.105009

[b24] Wang Y. *et al.* Differentiating characteristic microstructural features of cancerous tissues using Mueller matrix microscope. Micron 79, 8–15 (2015). 26280279 10.1016/j.micron.2015.07.014

[b25] Qi J., Ye M., Singh M., Clancy N. T. & Elson D. S. Narrow band 3 × 3 Mueller polarimetric endoscopy. Biomed. Opt. Express 4, 2433–2449 (2013). 24298405 10.1364/BOE.4.002433 PMC3829538

[b26] Kunnen B. *et al.* Application of circularly polarized light for non-invasive diagnosis of cancerous tissues and turbid tissue-like scattering media. J. Biophotonics 8, 317–323 (2015). 25328034 10.1002/jbio.201400104

[b27] Macdonald C. & Meglinski I. Backscattering of circular polarized light from a disperse random medium influenced by optical clearing. Laser Physics Letters 8, 324–328 (2011).

[b28] Macdonald C. M., Jacques S. L. & Meglinski I. V. Circular polarization memory in polydisperse scattering media. Physical Review E 91, 033204 (2015). 10.1103/PhysRevE.91.033204 25871235

[b29] Sankaran V., Walsh J. J. T. & Maitland D. J. Comparative study of polarized light propagation in biologic tissues. J. Biomed. Opt. 7, 300–306 (2002). 12175278 10.1117/1.1483318

[b30] Swami M. K. *et al.* Polar decomposition of 3 × 3 Mueller matrix: a tool for quantitative tissue polarimetry. Opt. Express 14, 9324–9337 (2006). 19529316 10.1364/oe.14.009324

[b31] Wang Y. *et al.* Study on the validity of 3 × 3 Mueller matrix decomposition. J. Biomed. Opt. 20, 065003–065003 (2015). 26039383 10.1117/1.JBO.20.6.065003

[b32] Manhas S. *et al.* Demonstration of full 4 × 4 Mueller polarimetry through an optical fiber for endoscopic applications. Opt. Express 23, 3047–3054 (2015). 25836165 10.1364/OE.23.003047

[b33] Collett E. Polarized light in fiber optics. (SPIE Press, 2003).

[b34] Ambirajan A. & Look D. C. Jr Optimum angles for a Mueller matrix polarimeter. Proc. SPIE 2265, 314–326 (1994).

[b35] Wood T. C. & Elson D. S. Polarization response measurement and simulation of rigid endoscopes. Biomed. Opt. Express 1, 463–470 (2010). 21258481 10.1364/BOE.1.000463 PMC3018013

[b36] Compain E., Poirier S. & Drevillon B. General and Self-Consistent Method for the Calibration of Polarization Modulators, Polarimeters, and Mueller-Matrix Ellipsometers. Appl. Opt. 38, 3490–3502 (1999). 18319949 10.1364/ao.38.003490

[b37] van Staveren H. J., Moes C. J. M., van Marie J., Prahl S. A. & van Gemert M. J. C. Light scattering in lntralipid-10% in the wavelength range of 400–1100 nm. Appl. Opt. 30, 4507–4514, (1991). 20717241 10.1364/AO.30.004507

[b38] Chipman R. A. Polarimetry. Handbook of optics 2, 1–28 (OSA, 1995).

[b39] Antonelli M.-R. *et al.* Mueller matrix imaging of human colon tissue for cancer diagnostics: how Monte Carlo modeling can help in the interpretation of experimental data. Opt. Express 18, 10200–10208 (2010). 20588874 10.1364/OE.18.010200

[b40] Alali S. *et al.* Quantitative correlation between light depolarization and transport albedo of various porcine tissues. J. Biomed. Opt. 17, 045004–045001 (2012). 22559678 10.1117/1.JBO.17.4.045004

[b41] Dunn A. & Richards-Kortum R. Three-dimensional computation of light scattering from cells. Selected Topics in Quantum Electronics, IEEE Journal of 2, 898–905 (1996).

[b42] Ghosh N. *et al.* Depolarization of light in a multiply scattering medium: Effect of the refractive index of a scatterer. Physical Review E 70, 066607 (2004). 10.1103/PhysRevE.70.066607 15697526

[b43] Bicout D., Brosseau C., Martinez A. S. & Schmitt J. M. Depolarization of multiply scattered waves by spherical diffusers: Influence of the size parameter. Physical Review E 49, 1767–1770 (1994). 10.1103/physreve.49.1767 9961398

[b44] Kim A. & Moscoso M. Influence of the relative refractive index on the depolarization of multiply scattered waves. Physical Review E 64, 026612 (2001). 10.1103/PhysRevE.64.026612 11497735

[b45] Guo X., Wood G., Ghosh M. F. N. & Vitkin I. A. Depolarization of light in turbid media: a scattering event resolved Monte Carlo study. Appl. Opt. 49, 153–162 (2010). 20062501 10.1364/AO.49.000153

[b46] Tuchin V. V., Wang L. V. & Zimnyakov D. A. Optical polarization in biomedical applications. (Springer, 2006).

[b47] Cotran R. S., Kumar V., Collins T. & Robbins S. L. Robbins pathologic basis of disease. (Saunders, 1999).

[b48] Jain R., Kasturi R. & Schunck B. G. Machine vision. Vol. 5. (McGraw-Hill, 1995).

[b49] Georgakoudi I. *et al.* NAD (P) H and collagen as *in vivo* quantitative fluorescent biomarkers of epithelial precancerous changes. Cancer Res. 62, 682–687 (2002). 11830520

[b50] Aitken K. J. & Bagli D. J. The bladder extracellular matrix. Part I: architecture, development and disease. Nat Rev Urol 6, 596–611 (2009). 19890339 10.1038/nrurol.2009.201

[b51] Wang L. V. & Wu H. Biomedical optics: principles and imaging. (Wiley-Blackwell, 2007).

[b52] Murakumo M. *et al.* Three-dimensional arrangement of collagen and elastin fibers in the human urinary bladder: a scanning electron microscopic study. The Journal of urology 154, 251–256 (1995). 7776441

[b53] Hadley K. C. & Vitkin I. A. Optical rotation and linear and circular depolarization rates in diffusively scattered light from chiral, racemic, and achiral turbid media. J. Biomed. Opt. 7, 291–299 (2002). 12175277 10.1117/1.1483880

[b54] Pierangelo A. *et al.* *Ex vivo* photometric and polarimetric multilayer characterization of human healthy colon by multispectral Mueller imaging. J. Biomed. Opt. 17, 066009–066001 (2012). 22734765 10.1117/1.JBO.17.6.066009

[b55] Agarwal N. *et al.* Spatial evolution of depolarization in homogeneous turbid media within the differential Mueller matrix formalism. Opt. Lett. 40, 5634–5637 (2015). 26625069 10.1364/OL.40.005634

[b56] Stoyanov D., Rayshubskiy A. & Hillman E. Robust registration of multispectral images of the cortical surface in neurosurgery. Paper presented at *9th IEEE International Symposium on Biomedical Imaging (ISBI)*, Barcelona. (IEEE, 2–5 May 2012). 10.1109/ISBI.2012.6235892.

[b57] Clancy N. T. *et al.* Multispectral image alignment using a three channel endoscope *in vivo* during minimally invasive surgery. Biomed. Opt. Express 3, 2567–2578 (2012). 23082296 10.1364/BOE.3.002567 PMC3469985

[b58] De Martino A., Garcia-Caurel E., Laude B. & Drévillon B. General methods for optimized design and calibration of Mueller polarimeters. Thin Solid Films 455, 112–119 (2004).

[b59] Laude-Boulesteix B., De Martino A., Drévillon B. & Schwartz L. Mueller polarimetric imaging system with liquid crystals. Appl. Opt. 43, 2824–2832 (2004). 15143805 10.1364/ao.43.002824

[b60] Goldstein D. & Goldstein D. H. Polarized Light. Vol. 83 (CRC Press, 2011).

