# Supplementary figures and images for: A high definition Mueller polarimetric endoscope for tissue characterisation
Source: Sci Rep. 2016 May 12;6:25953. doi: 10.1038/srep25953 (PMC4865982; doi:10.1038/srep25953)

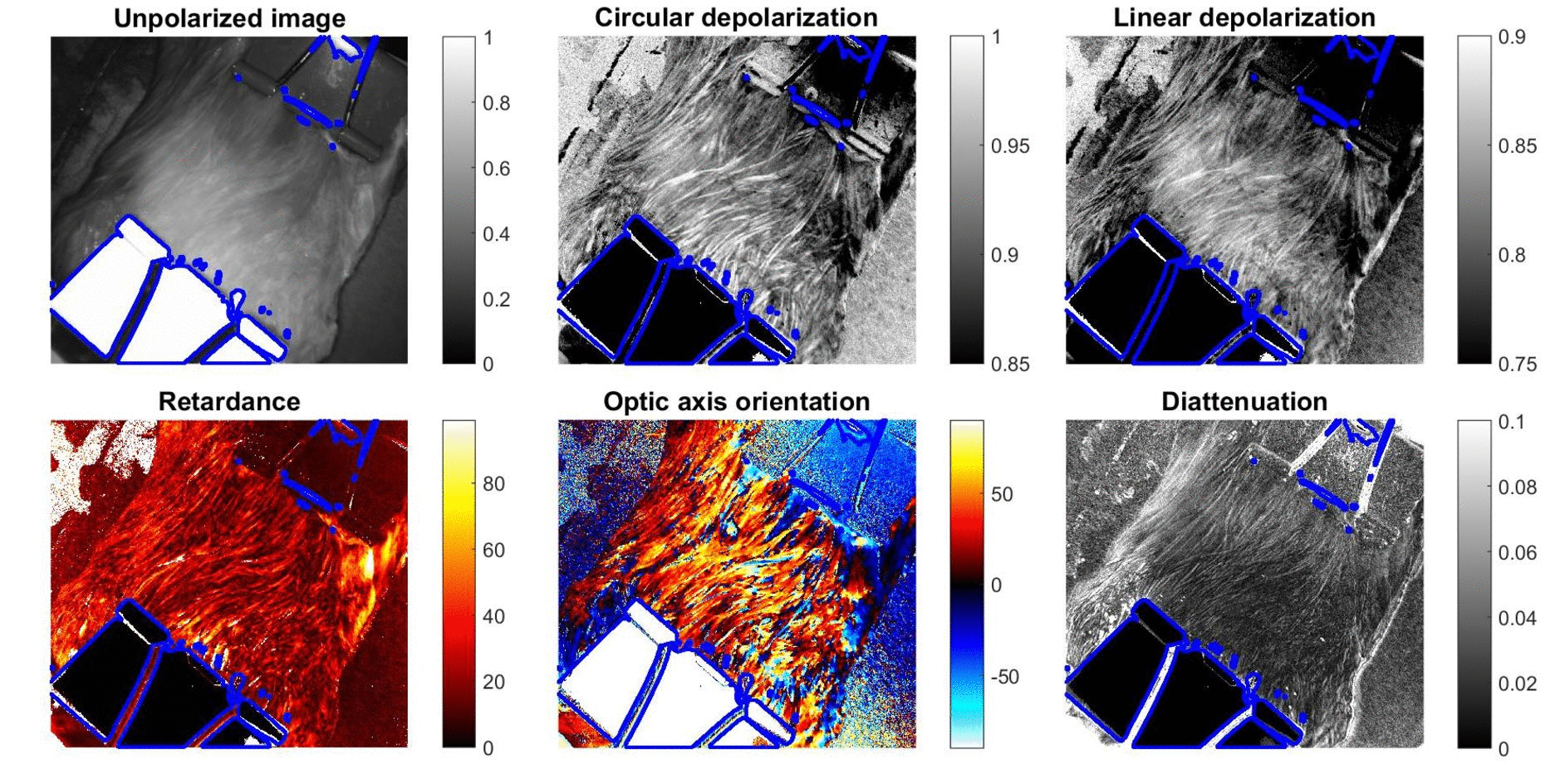

Supplement: Supplementary Video [file srep25953-s1.gif]
