# Supplementary material for: A high definition Mueller polarimetric endoscope for tissue characterisation
Source: Sci Rep. 2016 May 12;6:25953. doi: 10.1038/srep25953 (PMC4865982; doi:10.1038/srep25953)
Supplement: Supplementary Information [file srep25953-s2.pdf]

A high definition Mueller polarimetric endoscope for tissue characterisation

Ji Qi & Daniel S. Elson

1. Supplementary Video Legends

Experiments were performed on a porcine bladder to demonstrate that the MPE can reveal features that are either difficult to resolve or not resolvable for unpolarized endoscope by providing additional image contrast mechanisms. The bladder was continuously distended using two clamps, one of which was fixed while the other one was mounted on a linear translation stage oriented away from the first clamp. The displacement of the linear translation stage ranged from 0 to 28 mm with a step size of 1 mm and MPE images were recorded for each step. The acquired Mueller polarimetric images were decomposed into specific polarization images and shown in this video. The images in the first row are respectively unpolarized images, circular depolarization images and linear depolarization images. Those in the second row are retardance, optic axis orientation and diattenuation images respectively. Regions enclosed by the blue lines were invalid due to pixel saturation in at least one of the raw images for Mueller polarimetric image reconstruction.

2. Validation results

The validation results for the Mueller polarimetric endoscope are exhibited here. The objects imaged were a linear polarizer with extinction ratio 9000:1 (High contrast plastic linear polarizer, Edmund Optics Ltd. York, UK) orientated at 0° and 90°, and a circular polarizer obtained from a polarization-based 3D glasses (RealD Inc. California, USA) on the top of white paper. Paper has very rough surface and is a good depolarizer at normal incident with its Mueller matrix. The linear polarizers were mounted in a rotation stage. The circular polarizer had an irregular size mounted on a ring-shaped stage. Their 4×4 Mueller polarimetric images and the corresponding expected Mueller matrices were displayed in Figure 1, 2 and 3 respectively. Note that the circular polarizer also contained some linear diattenuation.

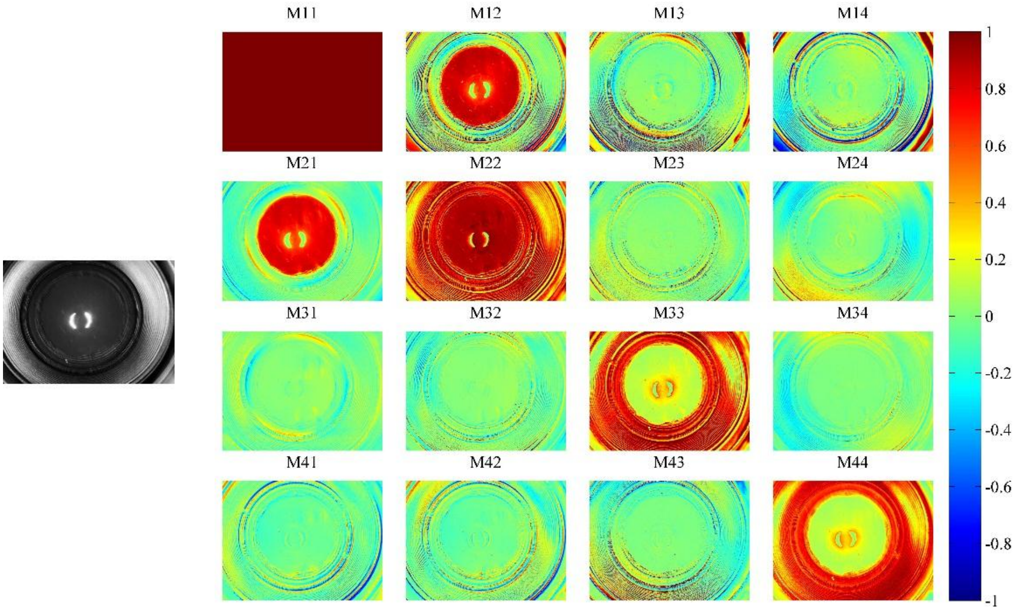

$$\begin{bmatrix} 1 & 1 & 0 & 0 \\ 1 & 1 & 0 & 0 \\ 0 & 0 & 0 & 0 \\ 0 & 0 & 0 & 0 \end{bmatrix}$$

(a) Unpolarized image (b) Mueller polarimetric image (c) Expected Mueller matrix

Figure 1. (a) The unpolarized image, (b) the Mueller polarimetric image and (c) the expected Mueller matrix of the linear polarizer orientated at 0°. The central circular part was the effective regions for imaging. The surrounding regions were the rotation stage for mounting purposes. The saturated regions in the centre of the field of view were caused by specular reflection.

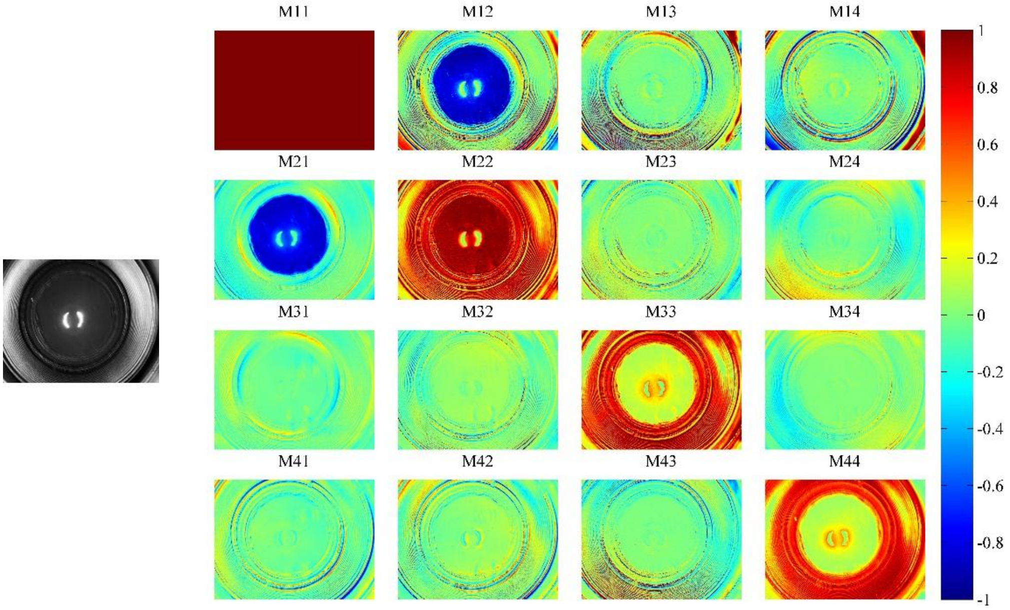

$$\begin{bmatrix} 1 & -1 & 0 & 0 \\ -1 & 1 & 0 & 0 \\ 0 & 0 & 0 & 0 \\ 0 & 0 & 0 & 0 \end{bmatrix}$$

(a) Unpolarized image (b) Mueller polarimetric image (c) Expected Mueller matrix

Figure 2. (a) The unpolarized image, (b) the Mueller polarimetric image and (c) the expected Mueller matrix of the linear polarizer orientated at 90°. The central circular part was the effective regions for imaging. The surrounding regions were the rotation stage for mounting purposes. The saturated regions in the centre of the field of view were caused by specular reflection.

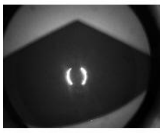

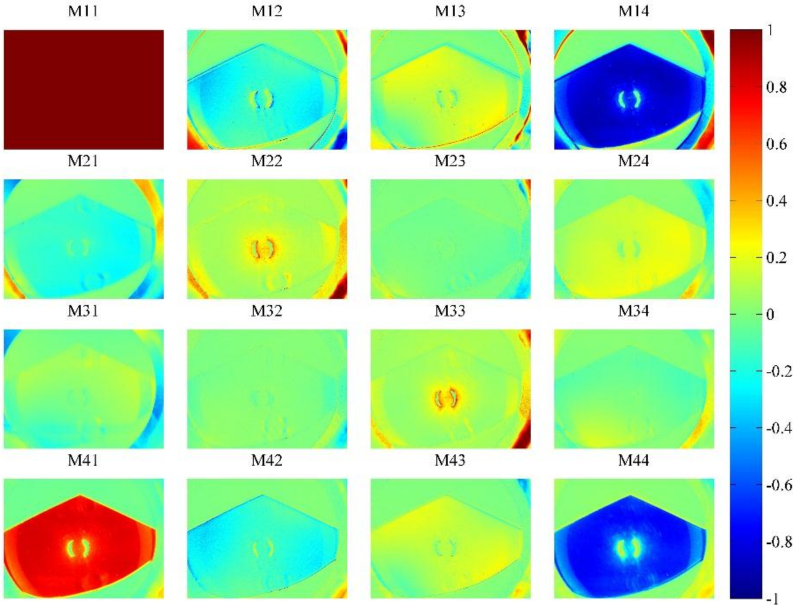

$$\begin{bmatrix} 1 & 0 & 0 & -1 \\ 0 & 0 & 0 & 0 \\ 0 & 0 & 0 & 0 \\ 1 & 0 & 0 & -1 \end{bmatrix}$$

(a) Unpolarized image (b) Mueller polarimetric image (c) Expected Mueller matrix

Figure 3. (a) The unpolarized image, (b) the Mueller polarimetric image and (c) the expected Mueller matrix of the linear polarizer orientated at 90°. The central circular part was the effective regions for imaging. The surrounding regions were the rotation stage for mounting purposes. The saturated regions in the centre of the field of view were caused by specular reflection.

3. Mueller polarimetric images of the tissue mimicking phantom and bladder

The Mueller polarimetric images of the tissue mimicking phantom and bladder were displayed in Figure 4-6. These images were then decomposed into retardance diattenuation and depolarization related images demonstrated in the paper. It is noted that the Mueller matrix images in Figure 4-6 were a normalised form of Mueller matrix (all the Mueller matrix elements have divided by M_{11}, except M_{11} itself). M_{11} corresponds to unpolarized radiometric image.

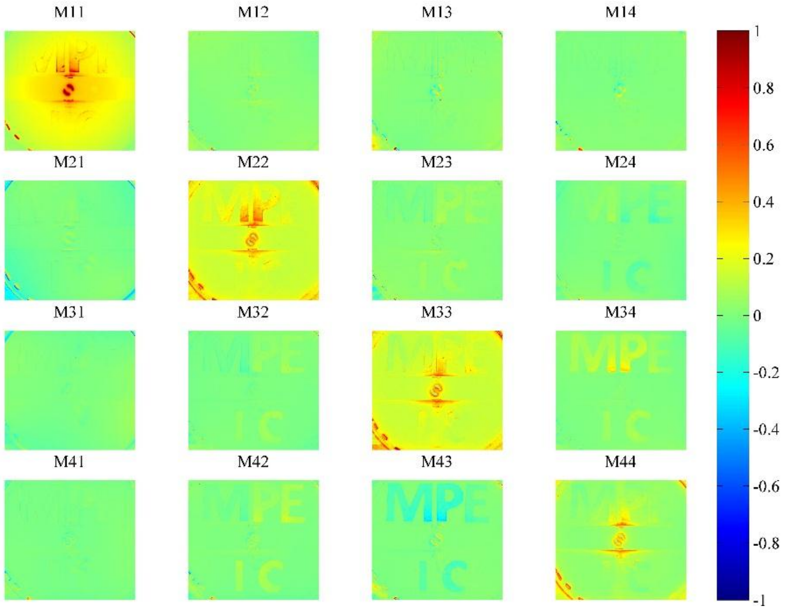

Figure 4. The Mueller polarimetric image of the tissue mimicking phantom shown in the paper.

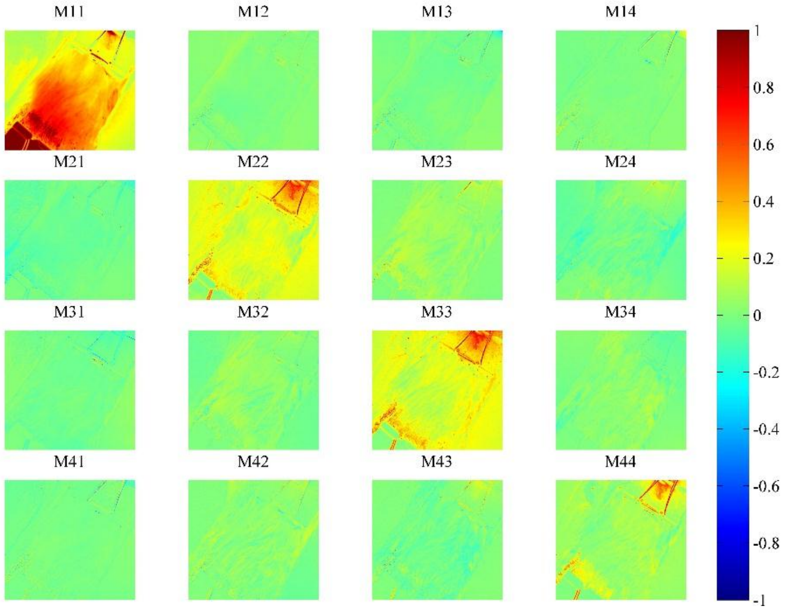

Figure 5. The Mueller polarimetric image of the bladder without distention.

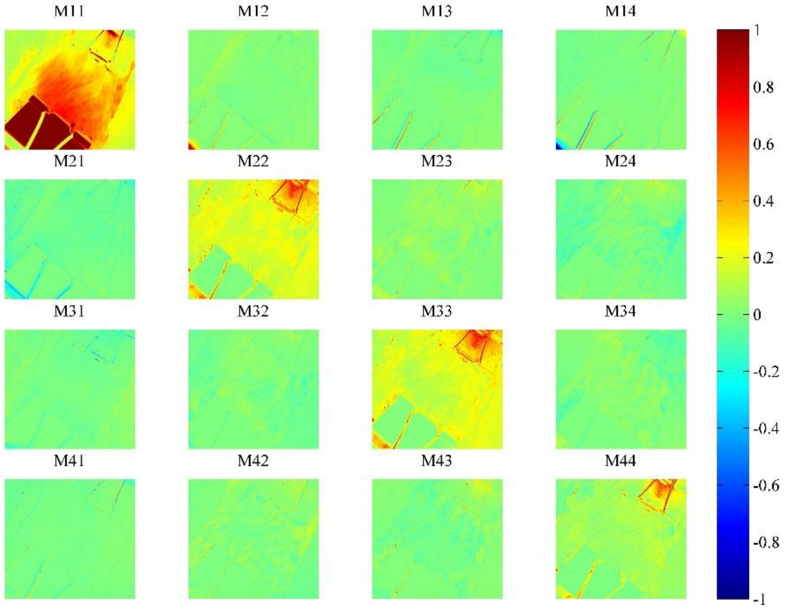

Figure 6. The Mueller polarimetric image of the bladder with distention.

4. Field of view, and digital resolution of the phantom and tissue experiments

The angle of view of this endoscopy system is about 60° as stated in “Results--/Mueller polarimetric endoscope system design and calibration--/the last paragraph” in the manuscript. This corresponds to about 9*9 cm^2 field of view with the working distance of the Mueller polarimetric endoscope about 8 cm. In the phantom and animal experiments, the working distance was about 8cm, and the regions near the edges of the field of view were empty and were therefore cropped: Figures 3 and 5 obtained from the animal experiment show 1.07 million pixels out of 1.4 million pixels, showing an area about 7.8*7.8 cm^2. Figure 2 obtained from the phantom experiment showed 0.75 million pixels showing an area about 6.5*6.5 cm^2.
